# Case report: A case of HIV seroconversion in a high-risk individual after 35 years of resistance with a CCR5Δ32 heterozygous genotype

**DOI:** 10.1016/j.virusres.2026.199770

**Published:** 2026-06-24

**Authors:** Najib Aziz, Eun-Young Kim, Beth D. Jamieson, Matthew J. Mimiaga, Jeremy Martinez, Roger Detels

**Affiliations:** a Department of Epidemiology, University of California Los Angeles, Los Angeles CA 90095, USA; b Department of Medicine, Northwestern University Feinberg School of Medicine, Chicago, IL 60011, USA,; c Department of Medicine, University of California Los Angeles, Los Angeles CA 90095, USA; d Department of Psychiatry & Biobehavioral Sciences, University of California Los Angeles, Los Angeles, CA, 90095, USA

**Keywords:** Antiretroviral, CCR5Δ32, CD4 T-cells, HIV, Infection, Receptor

## Abstract

Numerous studies have examined the factors contributing to the variation in disease progression among people living with HIV-1, identifying a range of genetic, immunologic and virologic factors that interact in a complex, multifactorial dynamic. However, investigating individual susceptibility to HIV-1 infection adds an additional layer of complexity. In this observational report, we focus on the case of a man (Case-2021) who previously remained HIV-1 negative despite 35 years of self-reported high-risk sexual behavior with other men while enrolled in the MACS/WIHS Combined Cohort Study (MWCCS). To understand why this individual stayed free of HIV-1 infection for so long and what led to his eventual seroconversion, we examined the available genetic, immunologic, and virologic laboratory data before and two weeks after his diagnosis of HIV-1 infection and initiation of antiretroviral therapy (ART). In addition, we looked into two other high-risk men from same group (HR-10) who have sex with men (MSM) enrolled in the MWCCS who seroconverted later in their enrollment. Furthermore, we performed similar laboratory tests to assess the effect of seasonal variations on blood biomarkers from the same clinical visit as Case-2021, including one individual who already was living with HIV-1 and another who has remained seronegative throughout their enrollment in the MWCCS. In conclusion, this Case-2021 observational report suggests that resistance to HIV-1 infection is likely multifactorial, involving high risk sexual behavior, HIV-1 variants, the genomic structure of the CCR5 receptor on CD4^+^ T cells, and age-related immune changes. Further study is needed to investigate both the genomic protective factors of CCR5 against HIV-1 infection and these age-related immune changes.

## Highlights

• In this observational case report, a man who self-reported high-risk sexual behavior sex with men (MSM) remained HIV negative since 1984. The last recorded negative of HIV-1 antibody test in the MACS/WIHS Combined Cohort Study (MWCCS) database was in May 2021.
• The man had never taken HIV PrEP, and had reported multiple sexually transmitted infections (STI) including gonorrhea and chlamydia in multiple anatomical sites (urethral, rectal and pharyngeal), CMV, and syphilis throughout the 35 years. In addition, he was positive for EBV, HSV-1, HSV-2 and negative for HBV and HCV.
• He tested HIV-1 antibody positive in the fall of 2021 and result of HIV AG/AB, 4th Generation, repeatedly reactive and his HIV-1 RNA, QN PCR (Quantitative real-time Polymerase Chain Reaction) was 19,100 copies/mL on December 2021.
• The presence of both HLA A*02 and HLA B*5701, together with the heterogeneous mutation CCR5 (WT/A32), may have provided a multifactorial genetic advantage to resisting full infection in this man.
• The conclusions of this observational report suggest that resistance to HIV infection is likely be multifactorial and may potentially influenced in part by the presence of CCR5 heterozygous genotype (WT/Δ32). A *unique immune system profile* of each individual could be dictating the infectivity and prognosis of HIV infection.

## Introduction

The complex interplay between acute HIV infection and the host immune response is not fully understood. It is known that the primary cellular receptor for HIV-1 entry is the CD4 molecule, which is expressed on T-cells and other immune cells, such as macrophages. HIV-1 also requires secondary co-receptors to bind and enter target cells. The two most common co-receptors, CCR5 and CXCR4, belong to the chemokine receptor family (Moore et al., 1997) and exhibit differential distribution across immune cell subsets. The majority of new infections are established in cells expressing CCR5, whereas CXCR4 is much less commonly utilized as a co-receptor and is typically targeted late in infection (Vicenzi et al., 2013). The presence of CD4 and CCR5 on both monocytes and activated/memory T cells allows HIV-1 to target and deplete CD4^+^ helper T cells and monocytes during both the acute and chronic phases of infection.

One clearly documented mechanism of resistance to HIV-1 infectivity depends on homozygosity for the delta-32 (a 32-base pair deletion) mutation in CCR5, which restricts strains of HIV-1 that require CCR5 to infect target cell (Liu et al., 1996; Marmor et al., 2001; O’Brien et al., 1997). Lack of CCR5 on CD4-expressing cells in individuals homozygous for this polymorphism (Δ32/Δ32) provides strong protection against HIV-1 infection (Michael et al., 1998; Gorry et al., 2002). HIV-1 infection in CCR5 Δ32 homozygous individuals is remarkably rare, with only eleven cases reported worldwide to date (Michael et al., 1998; Gorry et al., 2002). Zimmerman et al. suggested that white Caucasian men who have sex with men (MSM) with the homozygous CCR5 Δ32/Δ32 genotype are highly resistant to infection by CCR5-tropic HIV-1; conversely, infected white Caucasian men heterozygous for CCR5 (WT/Δ32) exhibit a slower rate of disease progression to AIDS (Zimmerman et al., 1997).

The effects of Δ32 heterozygosity on the immune response during acute HIV-1 infection remain poorly understood. However, the CCR5Δ32 heterozygous genotype (WT/Δ32) reduces the expression of functional CCR5 on the cell surface, which can provide slight protection against HIV-1 infection progression (Mohamed et al., 2022). The genetic and functional regulation of the primary HIV-1 co-receptor CCR5 may thus play a key role in promoting natural HIV-1 control (Ellwanger et al., 2020; Claireaux et al., 2022). Jaumdally et al. showed that HIV-1-exposed seronegative individuals, compared with HIV-1-unexposed controls, have lower systemic immune activation, reduced CCR5 expression on CD4+ ^+^T-cells, and lower levels of plasma cytokines (Jaumdally et al., 2017).

Moreover, Yang et al. studied the immunological profiles of three separate groups of individuals with different levels of exposure risk to HIV-1; The first group (HR10) consisted of 14 highly exposed seronegative MSM, 4 of whom were CCR5 Δ32 heterozygous. The second group (PTI) consisted of 7 seronegative MSM with evidence suggesting a possible transient HIV-1 infection without seroconversion, among whom only one was Δ32 homozygous. Lastly, the third group (blood bank donors) consisted of 14 low-risk individuals, two of whom were found to be Δ32 heterozygous (Yang et al., 2002).

## Case report

The three HIV-1 seroconverted individuals (Case-2002, Case-2005, and Case-2021) in this observational report were part of a highly exposed group of 14 HIV-1-exposed, uninfected men stratified into the top 10th percentile of sexual exposure risk for infection (HR10). Case-2002, Case-2005, and Case-2021 were born in 1957, 1958, and 1951, respectively. All three were White men who have sex with men (MSM) with baseline risk scores of 54, 54, and 51. These scores reflect the total number of anal-receptive exposures adjusted for condom usage frequency (classified as: no condom use, 100%; occasional, 80%; half the time, 50%; most of the time, 10%; always, 0%). By comparison, the overall HR10 group had a mean risk score of 104, roughly reflecting the number of unprotected anal-receptive sexual episodes over a 30-month period at the beginning of the Multicenter AIDS Cohort Study (MACS) (Yang et al., 2002). In his final health history assessment in April 2021, Case-2021 stated that he had never used pre-exposure prophylaxis (PrEP) and had reported 10 new partners for anal and oral receptive exposures. Longitudinal follow-up of the HR10 group within the MACS project revealed that three of the 14 MSM subsequently demonstrated HIV-1 seroconversion. The first participant (enrolled in 1984) seroconverted in October 2002 (Case-2002); the second (enrolled in 1985) seroconverted in August 2005 (Case-2005); and the third (enrolled in 1985) seroconverted in November 2021 (Case-2021). The presentation of Case-2021 raises the question of which factors allowed him to remain free of HIV-1 infection for up to 35 years before seroconversion, whereas Case-2002 and Case-2005 remained uninfected for 18 and 20 years prior to their respective seroconversions.

Genomic differences in immunologic parameters, including Human Leukocyte Antigen (HLA) typing and CCR5 genotypes, were reviewed among Case-2002, Case-2005, and Case-2021, all of whom were high-risk participants in the MACS.

The HLA typing data for Case-2002 and Case-2005 were negative for A02, B27, B35, and B57. Case-2002, who seroconverted in 2002, was heterozygous for the CCR5 deletion (WT/Δ32), while Case-2005, who seroconverted in 2005, possessed the homozygous CCR5 WT/WT genotype. At the time of seroconversion, Case-2002 had a CD4^+^T-cell percentage of 50%, and the mean relative fluorescence intensity (RFI) of the CD38 molecule on CD8^+^ T cells was 404. In contrast, Case-2005 had a CD4^+^ T-cell percentage of 19%, and the mean RFI of CD38 on CD8^+^ T cells was 5798. Prior to their seroconversion in 1998, the percentage of CCR5+ CD4^+^ T cells and the mean RFI of CCR5 on CD4^+^ T cells for the two cases were 20% (RFI: 3344) and 36% (RFI: 5320), respectively.

This observational report primarily focuses on Case-2021, who enrolled in the MACS in 1985 and underwent semiannual testing for HIV-1 antibodies. The last recorded negative HIV-1 antibody test in the MACS database for this participant occurred in May 2021. Historical data from a June 2019 visit showed a CD4^+^ T-cell percentage of 50% and a mean RFI of CD38 on CD8^+^ T cells of 404. Prior to 1998, his baseline percentage of CCR5+ CD4^+^ T cells was 22% with a mean CCR5 RFI on CD4^+^ T cells of 3472. The individual stated he was unsure when he contracted HIV-1, but he tested positive for HIV-1 antibodies in the fall of 2021. He visited his physician in November 2021, where laboratory results confirmed the HIV-1 infection. A 4th-generation HIV Ag/Ab assay was repeatedly reactive, and his plasma HIV-1 RNA quantitative real-time PCR (qPCR) was 19,100 copies/mL. In December 2021, he initiated a once-daily regimen of Biktarvy a combination antiretroviral therapy consisting of emtricitabine, tenofovir alafenamide, and bictegravir and has maintained adherence to this regimen up to the time of this writing.

In January 2022, Case-2021 visited the MACS clinic for a follow-up health history assessment and blood and urine collection. To better understand the potential contributors to his acquisition of HIV-1 after 35 years of exposure, we performed a comprehensive series of laboratory evaluations. At that time, Case-2021 had been taking Biktarvy for approximately two weeks, and his plasma HIV-1 RNA viral load had decreased to <20 copies/mL. To control for laboratory assay variability and seasonal variations in blood biomarkers, we selected two additional MACS participants who visited the clinic during the same month: one MSM living without HIV (seronegative control, SN) and one MSM living with HIV-1 (seropositive control, SP) who was stably suppressed on a Tivicay (dolutegravir) based antiretroviral regimen.

Case-2021 had never taken PrEP and reported a history of multiple sexually transmitted infections (STIs) over the 35-year period, including gonorrhea and chlamydia at multiple anatomical sites (urethral, rectal, and pharyngeal), cytomegalovirus (CMV), and syphilis. Additionally, he was seropositive for Epstein-Barr virus (EBV), HSV-1, and HSV-2, and seronegative for HBV and HCV.

Cryopreserved peripheral blood mononuclear cells (PBMC) and frozen plasma specimens from Case-2021 were sent to the laboratory of Dr. Steven Wolinsky at the Northwestern University Feinberg School of Medicine for genomic characterization. To determine the frequency of CD4^+^ T cells harboring proviral DNA, real-time *gag* qPCR was performed on genomic DNA isolated from 1 × 10⁶ PBMCs. The number of HIV-1 proviral DNA copies was determined to be 13 copies per 10⁶ PBMCs. No measurable HIV-1 RNA was detected in the plasma. The genomic DNA sample presented technical challenges during amplification and sequencing of the virus. However, the laboratory successfully amplified a partial fragment of the proviral *gag* sequence, which was subtyped as HIV-1 subtype B.

To quantify the frequency of cells harboring replication-competent HIV-1, a quantitative viral outgrowth assay (QVOA) was conducted, but it failed to detect replication-competent virus. Additionally, the laboratory attempted to activate the CD4^+^ T cells via ex vivo culture and treatment with the latency-reversing agent (LRA) AZD-5582 to induce viral expression from any latent reservoirs established prior to ART initiation. This yielded no success; the laboratory was unable to detect replication-competent viral RNA (vRNA) or proviral DNA from the stimulated cultures.

Evaluating viral tropism requires either a functional infection assay or sequencing of the V3 loop region of the *env* gene from isolated virus. Because the post-ART blood sample had a suppressed viral load of <20 copies/mL, the laboratory could not definitively determine the viral tropism of Case-2021. These findings support the hypothesis that early initiation of ART (Biktarvy), potentially synergizing with his heterozygous CCR5 WT/Δ32 genotype, efficiently suppressed further HIV-1 replication and infection of CD4^+^ T cells.

In addition to genotyping, we conducted a panel of laboratory tests for Case-2021 alongside the SN and SP controls. Whole blood or frozen PBMCs were utilized for cellular analytes, and frozen plasma was used for cytokine quantification. The results are presented in Table 1, Table 2. All analytes were measured according to the manufacturers' protocols in both the diagnostic reference and research laboratories.

**Table 1:** Percentages and absolute counts of lymphocyte subsets, complete blood count (CBC), standard lipid panel, comprehensive metabolic panel, and Hemoglobin A1c. All tests performed by Diagnostics reference laboratories.

**Cell Surface and blood cells markers**	**References**	**SN**^a^	**C-2021**^b^		**SP**^c^	
% CD3	57–85%	68	79		65	
Absolute CD3	840–3060 cell/μL	1247	1255		1706	
% CD4	30–61	44	38		**29**	
Absolute CD4	490–1740 Cell/μL	804	606		770	
% CD8	12–42	22	41		36	
Absolute CD8	180–1170 cell/μL	404	650		938	
CD4/CD8 Ratio	0.86–5.00	1.99	0.93		0.82	
White blood cell count (Thousand)	3.8–10.8 cell/μL	6.8	6.0		6.0	
Lymphocytes	850–3900 cell/μL	1775	1572		2652	
Monocytes	200–950 cell/μL	449	450		348	
Neutrophils	1500–7800 cell/μL	4386	3876		2862	
Red Blood Cell count (million)	4.20–5.80 cell/μL	4.72	4.91		5.33	
Hemoglobin	13.2–17.1 *g*/dL	13.8	15.2		14.5	
Hematocrit	38.5–50.0 %	40.2	43.5		42.8	
Platelet count (thousand)	140–400 cell /μL	405	160		273	
**Comprehensive metabolic panel, and Hemoglobin A1c.**						
Total Cholesterol	<200 mg/dL	178		207		182
HDL	>40 mg/dL	52		45		53
Triglycerides	<150 mg/dL	142		193		92
LDL	<100 mg/dL	102		129		110
Glucose	64–99 mg/dL	91		97		93
Hemoglobin A1c	<5.7%	5.6		5.2		5.4
Protein Total	6.1–8.1 mg/dL	7.2		6.8		7.2
Alkaline Phosphatase	36–130U/L	98		82		70
AST	10–40U/L	13		18		20
ALT	9–46U/L	9		15		15
GGT	3–90U/L	34		13		29

SN.

a : HIV-1 seronegative

b : HIV-1 seroconverter (C-2021)

c **:** HIV-1seropositive that seroconverted on 04/09/2014 and reported no male or female sexual partners at MWCCS visits in 2021- 2022.

**Table 2:** Plasma and level of HIV, serum levels of cytokines, INF-**γ** Elispot and HLA typing of Case-2021 along with HIV-1 seronegative control (SN) and HIV-1 seropositive control (SP).

**Plasma HIV-1 RNA, and serum antibody performed by Diagnostics laboratory**	**References value**	**SN**^a^	**C-2021**^b^	**SP**^c^
HIV 1 RNA Quantitative PCR	Non-detectable	TNP^d^	<20 det.^e^	<20 det.^e^
HIV 1/2 Ag/Ab,4th Gen test	Non-reactive	Non-reactive	Reactive	Reactive
**Serum level of cytokines with Luminex multiplex EIA performed by research laboratory of Dr. Epeldegui**				
IL-1β pg/mL	N/A	0.73	0.63	1.55
IL-6 pg/mL	N/A	6.50	2.76	1.22
IL-8 pg/mL	N/A	15.23	6.04	15.37
IL-10 pg/mL	N/A	1.01	2.56	0.96
TNF-α pg/mL	N/A	18.14	18.84	13.90
**Secretion of INF-γ after unstimulated and stimulated of PBMC with PHA^f^ or CEF**^g^**and Elispot EIA performed by research laboratory of MWCCS**				
Unstimulated PBMC (number of spot)	>0	1	1	0
PHA (5mg/ml) stimulated PBMC	N/A	64	85	137
CEF (16 mg/ml) stimulated PBMC	N/A	7	2	17
**HLA Genotyping (HLA Typing) performed by research laboratory of Dr. Steven Wolinsky**				
**HLA-A** A02		Positive	Positive	**N/A**
HLA-B B27		Negative	Negative	**N/A**
HLA-B B35		Negative	Positive	**N/A**
HLA-B B57		Negative	Positive	**N/A**
HLA-B B5701		Negative	Positive	**N/A**

SN.

a : HIV-1 seronegative.

b : HIV-1 seroconverter (C-2021).

c **:** HIV-1 seropositive.

d : Test not performed, det.

e **:** detected, f: PHA: Phytohemagglutinin.

g **:** Cytomegalovirus**,** Epstein-Barr Virus and Influenza virus peptides pool. Case 2021 was heterozygous for CCR5 (WT/Δ32) with 22% of his CD4+ cells expressing CCR5 for a mean RFI of 3472.

## Discussion

The results of the blood panel for the HIV-1 seronegative control (SN), seropositive control (SP), and Case-2021 are presented in Table 1, Table 2. The percentage results of basic lymphocyte phenotypes for Case-2021 show increases (indicated in red) and decreases (indicated in blue) for certain markers relative to the two controls (Table 1); however, this small sample size provides no clear-cut insight into the reasons for late seroconversion. Furthermore, we did not expect to observe high levels of plasma cytokines following antiretroviral therapy (ART) in blood collected during Case-2021′s January 2022 visit, given the known suppression effect of the CCR5 WT/Δ32 genotype on HIV-1 infection of CD4^+^ T cells. Additionally, accurately interpreting these biomarkers is problematic due to the small sample size. Visualizing the differences in plasma levels of cytokines IL-1β, IL-6, IL-8, IL-10, and TNF-α among Case-2021 and the two controls relies on observational data (Table 2). These differences may be caused by low cytokine levels in the individual's blood circulation, or by the low sensitivity and minimum detection limits of the assay kit.

We observed variation in Human Leukocyte Antigen (HLA) typing between two other high-risk MACS participants, Case-2002 and Case-2005. While Case-2002 is heterozygous for the CCR5 deletion (WT/Δ32), Case-2005 is CCR5 wildtype homozygous (WT/WT) and should therefore have no overt barriers preventing CCR5-tropic viruses from infecting his CD4^+^ T cells. HLA typing for both cases revealed they were negative for HLA-A02, B27, B35, and B57, meaning neither possessed known genetic advantages in their immune response to HIV-1. Following seroconversion, the high-risk Case-2002 maintained a CD4^+^ T-cell percentage of 50%, whereas Case-2005 exhibited a similar high-risk profile but a CD4^+^ T-cell percentage of only 19%. Prior to their respective seroconversion visits, the percentage of CCR5+ CD4^+^ T cells and the mean RFI of CCR5 on CD4+ T cells were 20% and 3344 for Case-2002, compared to 36% and 5320 for Case-2005. This allows for speculation that Case-2005 may have lost a greater number of CD4^+^ T cells due to more efficient HIV-1 entry into CD4-expressing cells, while Case-2002 was protected by lower amounts of cell-surface CCR5. Unlike Cases 2002 and 2005, Case-2021 is HLA-A02 positive. HLA-A*02 appears to stimulate peripheral blood mononuclear cells in a manner that confers resistance to HIV-1 replication. Prior publications demonstrate that HLA-A02-positive infants have a documented nine-fold reduction in HIV transmission during childbirth (Grene et al., 2001). Furthermore, HLA-B57 molecules are highly polymorphic. The most frequently expressed HLA-B57 alleles are HLA-B*57:01, HLA-B*57:02, and HLA-B*57:03; among these, HLA-B*57:01 and HLA-B*57:03 are associated with HIV-1 control in Caucasian and African individuals, respectively. Individuals carrying a protective HLA-B*57 allele experience slower HIV-1 disease progression, often maintaining undetectable plasma viral loads and normal CD4^+^ T-cell counts without medication (Brennan et al., 2012; Gillespie et al., 2006; Lobos et al., 2022). Case-2021 was heterozygous for HLA-B*57:01 (Table 2).

The central question remains: what factors enabled Case-2021 to remain uninfected with HIV-1 for 35 years, while failing to provide the same long-term protection to Case-2002 and Case-2005, who seroconverted after 18 and 20 years, respectively?

Ultimately, a complex combination of immunological profiles, genomic variations, HLA typing, and environmental factors likely dictates the infectivity and prognosis of HIV infection. Case-2021 may have benefited from a synergistic interplay of these variables to evade HIV-1 infection for 35 years, in contrast to Case-2002 and Case-2005.

Our observational report has notable limitations, including a small sample size that limits statistical power. Additionally, suboptimal timing of blood collection from the ART-treated Case-2021 may have impacted the measured blood levels of cytokines, cellular markers, and plasma viral loads during the early stages of HIV-1 infection.

## Conclusion

The concurrent presence of HLA-A*02, HLA-B*57:01, and the heterozygous CCR5 WT/Δ32 mutation likely provided a multifactorial genetic advantage that helped Case-2021 resist full infection. Finally, what tipped the scales to trigger seroconversion at that specific moment remains unclear and raises several questions. Advancing age diminishes immune responses, and unusual immune activation at the time of exposure—or infection with a specific HIV-1 variant—may have played a role. Further longitudinal studies are needed to evaluate the protective mechanisms of CCR5 mutations and the impact of immunosenescence on HIV-1 susceptibility.

## Consent for publication

The institutional review board (IRB) for human studies at UCLA (University of California, Los Angeles) approved the protocols. All participants signed an informed consent.

## Funding

The contents of this publication are solely the responsibility of the authors and do not represent the official views of the National Institutes of Health (NIH). MWCCS (Principal Investigators): Los Angeles CRS (Roger Detels and Matthew Mimiaga), U01-HL146333; Data Analysis and Coordination Center (Gypsyamber D’Sousa, Stephen Gange and Elizabeth Topper), U01-HL146193.The MWCCS is funded primarily by the National Heart, Lung, and Blood Institute (NHLBI), with additional cofounding from the Eunice Kennedy Shriver National Institute of Child Health & Human Development (NICHD), National Institute on Aging (NIA), National Institute of Dental & Craniofacial Research (NIDCR), National Institute of Allergy And Infectious Diseases (NIAID), National Institute of Neurological Disorders and Stroke (NINDS), National Institute of Mental Health (NIMH), National Institute on Drug Abuse (NIDA), National Institute of Nursing Research (NINR), National Cancer Institute (NCI), National Institute on Alcohol Abuse and Alcoholism (NIAAA), National Institute on Deafness and Other Communication Disorders (NIDCD), National Institute of Diabetes and Digestive and Kidney Diseases (NIDDK), National Institute on Minority Health and Health Disparities (NIMHD), and in coordination and alignment with the research priorities of the National Institutes of Health, Office of AIDS Research (OAR). MWCCS data collection is also supported by UL1-TR001881 (UCLA CTSI)

## CRediT authorship contribution statement

**Najib Aziz:** Writing – review & editing, Writing – original draft, Conceptualization. **Eun-Young Kim:** Writing – review & editing, Validation, Methodology, Formal analysis. **Beth D. Jamieson:** Writing – review & editing, Validation, Investigation. **Matthew J. Mimiaga:** Writing – review & editing, Investigation, Funding acquisition. **Jeremy Martinez:** Writing – review & editing, Visualization, Resources. **Roger Detels:** Writing – review & editing, Supervision, Investigation, Funding acquisition.

## Declaration of competing interest

All co-authors including corresponding author declare no conflict of interest with this paper.

## Data Availability

Data will be made available on request.
